# Evaluation of a Scalable Design for a Pediatric Telemedicine and Medication Delivery Service: A Prospective Cohort Study in Haiti

**DOI:** 10.4269/ajtmh.24-0846

**Published:** 2025-06-24

**Authors:** Molly B. Klarman, Xiaofei Chi, Youseline Cajusma, Katelyn E. Flaherty, Jude Ronald Beausejour, Lerby Exantus, Valery Madsen Beau de Rochars, Chantale Baril, Torben K. Becker, Matthew J. Gurka, Eric J. Nelson

**Affiliations:** ^1^ Department of Pediatrics, University of Florida, Gainesville, Florida;; ^2^ Department of Environmental and Global Health, University of Florida, Gainesville, Florida;; ^3^ Department of Emergency Medicine, University of Florida, Gainesville, Florida;; ^4^ Faculté de Médecine et de Pharmacie, Université d’État d’Haiti, Port-au-Prince, Haiti;; ^5^ Department of Health Services Research, Management and Policy, University of Florida, Gainesville, Florida;; ^6^ Department of Public Health Sciences, University of Virginia, Charlottesville, Virginia

## Abstract

Early access to health care is essential to avert morbidity and mortality. A telemedicine and medication delivery service (TMDS) is an innovative solution to address this need; however, pathways to scalability are unclear. We sought to evaluate a scalable pediatric TMDS. A TMDS in Haiti was configured for scalability by triaging severe cases to hospital-level care, nonsevere cases with higher clinical uncertainty to in-person examinations at households, and nonsevere cases with low clinical uncertainty to medication delivery alone. This design was evaluated in a prospective cohort study conducted among pediatric patients 10 years old or younger. Clinical and operational metrics were compared with a formative reference study in which all nonsevere patients received an in-person examination. The primary outcomes were rates of clinical improvement/recovery and in-person care seeking at 10 days. In total, 1,043 cases were enrolled in the scalable TMDS mode, and 19% (190) of nonsevere cases received an in-person examination; 382 cases were enrolled in the reference study, and 94% (338) of nonsevere cases received an in-person examination. At 10 days, rates of improvement were similar for the scalable and reference modes. Rates of participants who sought follow-up care were 15% in the scalable mode and 24% in the reference mode. In the context of a 5-fold reduction of in-person examinations, participants in the scalable mode had noninferior rates of improvement at 10 days. These findings highlight an innovative and now scalable solution to improve early access to health care without compromising safety.

## INTRODUCTION

The WHO established the Integrated Management of Childhood Illness (IMCI) guidelines for the management of sick children in 1995.^1^^,^^2^ The approach has since been deployed globally and is the current standard for community-based pediatric care in low- and middle-income countries.^3^ There are numerous challenges to the successful implementation of the IMCI strategy that limit its potential to effectively reduce morbidity and mortality.^4^^–^^8^ Implementation science frameworks have facilitated the design, evaluation, and uptake of potential solutions to address these complex challenges.^9^ Solutions include technologies aimed at bridging the “know-do gap,” specifically the gap between knowledge of the IMCI guidelines and effective implementation of the guidelines.^10^^–^^14^

Despite these advances, there are gaps in the accessibility of IMCI-adherent clinics. We seek to close these gaps by extending early access to high-quality pre-emergency pediatric health care at the household level. We focus on the nighttime when health care seeking is often delayed to the morning because of financial and logistical barriers.^15^^,^^16^ Delays increase the risk of developing life-threatening disease, which can lead to higher-cost interventions.^17^^,^^18^ In response, we designed and implemented a pediatric telemedicine and medication delivery service (TMDS), called MotoMeds, in Haiti that uses clinical resources derived from the IMCI guidelines.

Reliable health and demographic data have become increasingly difficult to obtain in Haiti because of the ongoing political and security instability. Haiti has historically had one of the lowest health care workforce densities in the world. As of 2018, there were 0.2 physicians and 0.4 nurses/midwives per 1,000 people.^19^ These figures have worsened because of high emigration rates among health care professionals. By 2024, the United Nations Children’s Fund estimated that 40% of the country’s health care workforce had left because of insecurity.^20^ Approximately 60% of the country’s 11.5 million population once resided in the capital, Port-au-Prince. However, an estimated 261,000 people have been displaced since 2021.^21^^,^^22^ The capital hosts the majority of the nation’s teaching hospitals and advanced medical facilities, yet many of these institutions have either closed or become prohibitively difficult to access because of security concerns.^23^ Additionally, the capital has traditionally been the center of trade and economic activity. However, the escalating security crisis has disrupted supply chains and impacted the delivery of essential services across the island.^24^ Despite 90% of the population having access to the internet through smartphones, the reliability and availability of service remain substandard because of fuel shortages and disruption in equipment maintenance caused by restricted movement.^25^

The TMDS model was developed and rigorously evaluated within the Improving Nighttime Access to Care and Treatment (INACT) study series. The INACT studies began in Haiti and expanded to Ghana to test adaptability.^15^^,^^16^ In Haiti, the INACT1-H study characterized care-seeking barriers.^15^ The INACT2-H study was a prepilot that established clinical safety, feasibility, and guideline performance of a TMDS.^26^^,^^27^ The study tested if “what was heard” over the phone per virtual examinations matched “what was seen” at the bedside per in-person examinations. Severe cases were referred to hospital-level care. All nonsevere cases received paired virtual and in-person examinations to determine the accuracy of the virtual assessments and treatment plans. This study identified high rates of improvement/recovery at 10 days (95%). The evaluation of most nonsevere cases had what we considered low clinical uncertainty (see the Materials and Methods section). Based on these results, we hypothesize herein that these cases can be safely managed with medication delivery alone. However, we recognized that a subset of cases had higher clinical uncertainty, and we reasoned that these cases will continue to require in-person evaluation. Our overall rationale is that configuring the TMDS to align with our hypothesis will reduce in-person examinations by approximately 85% without compromising the high rates of improvement/recovery.

Therefore, the INACT3-H study presented here aims to test our clinical guidelines, guiding hypothesis, and overall approach as a pathway to scalability. Within implementation science frameworks for scaling,^28^ our approach addresses the facilitator: “designing the innovation for scale.”^29^^,^^30^ In doing so, the primary design modification was removing the clinical “guardrail” of in-person examinations for nonsevere cases with low clinical uncertainty. A secondary design modification was restructuring the TMDS into a “hub-and-spoke” model, where one central call center serviced multiple delivery zones. These modifications are anticipated to reduce the financial and human resources required to operate a TMDS.^31^ Our approach was respectful of the challenges with real-world implementation of novel innovations, and the study protocol was permissive to iterations. This “scalable” design was implemented within a prospective cohort study (INACT3-H), and the results were compared with the previously published prospective cohort study as a reference standard (INACT2-H).

## MATERIALS AND METHODS

### Study design.

We sought to evaluate a scalable design for a pediatric TMDS by comparing outcome measures from the scalable mode with a prior reference mode. The INACT2-H and INACT3-H studies were prospective cohort studies. The INACT2-H workflow was modified to facilitate scalability.^26^ The scalable mode, defined below, was deployed in the INACT3-H study. Outcomes from INACT3-H were compared with those from INACT2-H (reference standard).

### Study population and setting.

INACT2-H was conducted in Gressier, Haiti from September 9, 2019 to January 19, 2021. INACT3-H was conducted in Gressier from January 20, 2021 to September 21, 2022 and in Les Cayes from October 6, 2021 to September 21, 2022. Gressier has a population of 36,400 and is characterized by rural mountainous regions and more densely populated coastal communities.^32^ Les Cayes is approximately 175 km west of Gressier. It has a population of 152,000 people, and over half live in the urban center.^32^ On August 14, 2021, Les Cayes was impacted by a 7.2-magnitude earthquake that displaced inhabitants and impacted population dynamics.^33^ The delivery zone in Les Cayes was added to respond to the humanitarian need after the earthquake. Both Gressier and Les Cayes have minimal public infrastructure (e.g., electricity, paved streets, and reliable telecommunication services). The study periods were marked by spikes of political and societal instability that impacted the movement of goods and provision of services with detrimental effects on health and health care.^34^^,^^35^

### Participant recruitment.

Participant populations were informed of the TMDS through radio and print advertisements as well as announcements at schools and clinics.

### Participant inclusion criteria and consent process.

For both studies, children were eligible to participate if their parent contacted the TMDS during operational hours (6 pm to 5 am) regarding an illness experienced by their child 10 years old or younger. Written consent from parents/guardians and assent from children 7 years old or older were obtained from families who received an in-person examination. Parents/guardians whose only contact with the TMDS was a virtual encounter were read a waiver of documentation of consent over the phone.

### Enrollment.

Enrollment estimates for the scalable mode (INACT3-H) were based on expected call volume that was established in the reference mode (INACT2-H), which generally varied between one and three calls per night. Enrollment during INACT3-H was increased iteratively to assure that both research and service objectives were met in response to dynamic national safety and logistical challenges.

### Participant incentives and fees.

Across both studies, public messaging conveyed that the service was available for a nominal fee. Upon completion of virtual examinations, TMDS users whose treatment plans included medication delivery were asked to contribute 500 Gourdes (5 U.S. dollars) toward the associated costs. If a parent indicated an inability to pay, they were informed that the fee could either be reduced or be waived.

### Implementation.

#### TMDS workflow.

**Figure 1.**
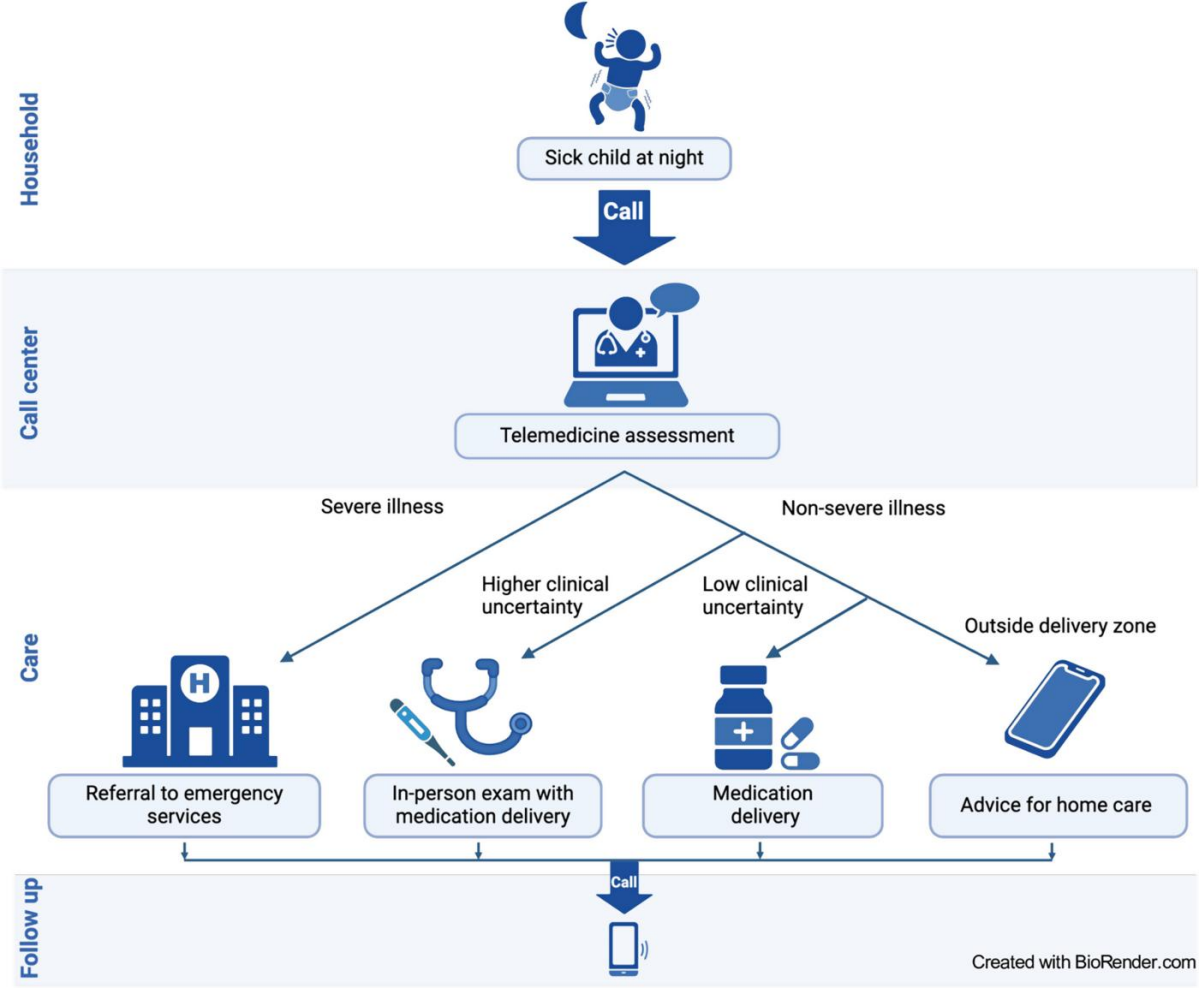
General workflow of a telemedicine and medication delivery service (TMDS). The parent of a child who is sick at night contacts the call center. A TMDS provider receives the call and completes a virtual examination by asking the parent targeted questions about the child and their symptoms. The provider references clinical resources to create a treatment plan based on illness severity that includes disposition, medications/fluids, and follow-up recommendations. Families are contacted by phone at 24 hours and/or 10 days. In the reference mode (Improving Nighttime Access to Care and Treatment 2-H), all nonsevere cases residing within the delivery zone received a paired in-person examination at the household for research purposes, and medication delivery (alone) was not part of the workflow.^19^

The workflow elements shared between the studies were as follows (Figure 1). 1) A parent contacted the call center and was connected to a provider. 2) The provider gathered demographic and clinical information about the sick child, referenced clinical resources, and generated a treatment plan that included triage classification (mild, moderate, or severe), treatment location (hospital or home), recommended medications and/or fluids, and advice on follow-up care. 3) Severe cases were immediately referred to the hospital. 4) For nonsevere cases, those residing in a delivery zone received an in-person examination by a provider and/or delivery of medications/fluids. 5) Children residing outside a delivery zone or who did not require medications/fluids received virtual advice alone. 6) Patients requiring hospital or mandatory clinic follow-up received a 24-hour follow-up call. All patients received a 10-day follow-up call to ascertain the child’s condition and feedback on the service.

#### TMDS elements specific to the reference mode (INACT2-H).

The call center served a single delivery zone in Gressier, Haiti. Call center providers performed in-person examinations for all nonsevere cases within the delivery zone; the paired examinations were used to assess the accuracy of the virtual examinations.

#### TMDS workflow elements specific to the scalable mode (INACT3-H).

The INACT2-H call center and the Gressier delivery zone were maintained and a second delivery zone in Les Cayes, Haiti was added in response to humanitarian need caused by an earthquake. The TMDS workflow was configured to reserve in-person examinations for nonsevere cases with higher clinical uncertainty. Participant siblings of these cases also received in-person examinations to refine their treatment plans and to collect additional paired virtual/in-person examination data. For nonsevere cases with low clinical uncertainty, delivery drivers were dispatched to transport medications/fluids to participant households. We estimated that this change would limit in-person examinations to approximately 15% of all cases. In the “delivery-alone” situations, instructions on how to prepare and administer the medications/fluids were relayed by the provider to the parent over the phone at the end of the virtual examination. The information was reinforced through video tutorials that were played for parents on a tablet at the household; parents were offered an opportunity to reconnect to call center providers for clarifications.

### Provider and staff training.

The providers were licensed Haitian nurses and nurse practitioners. Six of 10 call center providers worked for the TMDS during both the INACT2-H and INACT3-H studies. Licensed Haitian physicians were available by phone to provide advice on cases outside the scope of the clinical resources. All providers and drivers received didactic and experiential training relative to their clinical, research, and operational responsibilities.

### Clinical procedures, guidelines, and resources.

Providers were equipped with three primary clinical resources (Mendeley Data, v. 1, doi: 10.17632/7924gg3t9z.1) to guide the virtual and in-person examinations. These resources are described elsewhere and include the following.^27^ The first resource was clinical guidelines based on in-person WHO pediatric resources and adapted for telemedicine use. The guidelines prioritized six common childhood illnesses/problems: “fever,” “respiratory problem/cough,” “dehydration/vomit/diarrhea,” “ear pain,” “skin problem,” “pain with urination,” and “other.” Each section began with a summary of the problem and was stratified by the diagnostic criteria used to triage the severity of the problem into mild, moderate, or severe categories. Criteria that designated a case as severe were referred to as “danger signs.” Within each stratum, there was guidance on how to treat, where to treat, and when to follow-up. Directives on treatment location were based on severity and clinical uncertainty. Regarding “clinical uncertainty,” a strict definition is difficult to set and build into guidelines because it is the collective impression that the provider establishes based on both objective and subjective data. Our guidelines were derived from IMCI guidelines, which adhere to broad standards of pediatric care and organically make recommendations based on degrees of uncertainty. With this mindset, our guidelines prioritized in-person examinations for patients younger than 1 year old, especially those with fever and/or concern for fever and cough, as well as patients with concern for dehydration (e.g., 12+ episodes of diarrhea) or concerns for respiratory insufficiency (e.g., fast breathing). These conditions are associated with high clinical uncertainty as they often present subtly yet are vulnerable to progressive decompensation, specifically from sepsis, pneumonia, and hypovolemic shock. The second resource was a paper case report form that guided data collection during virtual and in-person examinations and was embedded with clinical decision support. The third resource was a medication formulary that outlined available medications/fluids and dosing recommendations. These resources have been iterated and adapted to real-world use since they were first piloted in September 2019. The resources were written for midlevel health care providers (e.g., experienced nurses, nurse practitioners). Although the guidelines did not explicitly state when a case scenario has higher versus low clinical uncertainty, uncertainty is built into the algorithms, which follow WHO standards of practice.^2^^,^^36^ The WHO standards are based in part on risk stratifications set by consensus-based expert opinion or evidence-based primary clinical research studies. The clinical guidelines and resources remain intended for use in this INACT study series alone.

### Outcome measures.

#### Primary.

The primary outcome measures were rates of improvement/recovery and rates of in-person care seeking at the 10-day follow-up.

#### Secondary.

Secondary outcome measures consisted of operational, performance, and 10-day feedback components. Operational measures were “virtual examination duration” defined as the length of time to complete a virtual examination over the phone and “time to arrival at household” defined as the length of time spanning from initial contact with the TMDS by phone until a driver (with or without a provider) arrived at the participant’s house. Performance measures focused on congruence and guideline deviations. Congruence refers to the agreement between components of the paired virtual and in-person examinations; congruence was assessed for case severity; presence of fever, fast breathing, and dehydration; and treatment with select medications. Guideline deviations were instances when a provider made a clinical decision outside the scope of the written clinical guidelines. Deviations were expected and may have occurred because of human error, situational adaptability, or direction from the oversight physician. Deviations were quantified for danger signs and select medications provided. User feedback was obtained from families in structured (Likert scale) and unstructured (free text) formats during the 10-day follow-up phone call.

#### Exploratory.

Analyses were stratified by type of care provided and delivery zone location.

### Data analysis.

Clinical, operational, and performance outcome measures were compared between the scalable (INACT3-H) and reference (INACT2-H) modes.

### Analytic strategy.

Repeat cases within 30 days were excluded from both study cohorts. Participants not reached at the 10-day follow-up were excluded from the clinical outcome and feedback analyses. Missing data for other variables were addressed through pair-wise deletion. In the scalable mode, guideline deviations were identified by internally reviewing the data for instances of missed danger signs and accuracy of medications included in treatment plans (e.g., complaints of pus in ear should yield inclusion of amoxicillin in plan). The identification of guideline deviations occurring in the reference mode was described previously.^26^ When calculating guideline deviation rates, the number of completed examinations (virtual and in person) was used as the unit of analysis. Congruence was assessed for the subset of cases that had paired virtual and in-person examinations, which mainly included cases with higher clinical uncertainty. Comparisons that involved a guideline deviation were excluded from the respective congruence analyses. The subset of medications that were included in the congruence analyses were selected because of their centrality to the clinical guidelines and were provided most frequently.

## STATISTICAL ANALYSES

Analyses were completed using Statistical Analysis Software v. 9.4 (SAS Inc., Cary, NC). Proportions were used to describe categorical variables, whereas medians with interquartile ranges (IQRs) were used to describe continuous variables. To test differences between the modes (scalable and reference) or between locations within the scalable mode (Gressier and Les Cayes), χ^2^ tests or Fisher exact tests were used for nominal variables, Cochran–Armitage trend tests were used for ordinal variables, and two-sample *t*-tests were used for continuous variables. To compare clinical and operational outcomes between the two modes stratified by type of care provided, Cochran–Mantel–Haenszel tests (general association) were used for nominal variables, Cochran–Mantel–Haenszel tests (row mean scores differ) were used for ordinal variables, and two-way analyses of variance were used for continuous variables. Binary assessments were described using Cohen’s kappa, sensitivity, specificity, positive predictive values (PPVs), and negative predictive values (NPVs). Assessments with kappa values were classified as follows: −0.1 to 0.20 indicates “no agreement,” 0.21–0.39 indicates “minimal,” 0.40–0.59 indicates “weak,” 0.60–0.79 indicates “moderate,” 0.80–0.90 indicates “strong,” and >0.90 indicates “almost perfect.”^37^

## RESULTS

### Participant and case characteristics.

**Figure 2.**
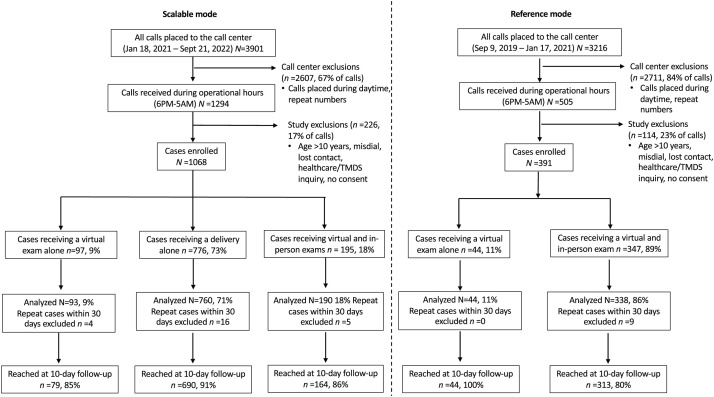
Enrollment. Diagram of case enrollment and inclusion in analyses for scalable and reference telemedicine and medication delivery service modes. TMDS = telemedicine and medication delivery service.

The reference mode enrolled 391 participants, and the scalable mode enrolled 1,068 participants. Among enrolled participants, 1,043 from the scalable mode and 382 from the reference mode were included in the analyses (Figure 2). The median age was 24 months old (IQR: 12–48 scalable; 9–48 reference) for both cohorts, and sex distributions were similar (Table 1). There was a lower proportion of participants younger than 2 months old in the scalable cohort (1%, *n* = 15) compared with the reference cohort (7%, *n* = 26). The chief and general complaints were similar among both modes. The most prevalent chief complaint was “fever” (33%, 342 scalable; 42%, 159 reference) followed by “respiratory problem” (24%, 247 scalable; 17%, 67 reference), whereas the most prevalent general complaint was “respiratory problem” (66%, 686 scalable; 65%, 247 reference) followed by “fever” (55%, 576 scalable; 55%, 212 reference). Virtual complaints of “fever” were determined subjectively in all but seven cases (one, 1% scalable; six, 3% reference). Gastrointestinal and dermatologic complaints were also common. Based on the virtual examinations, approximately three fourths of cases were triaged as mild in both the scalable (76%, *n* = 788) and reference (73%, *n* = 278) modes. In the scalable mode, 18% (*n* = 190) of cases received a virtual examination and an in-person examination with delivery, and 73% (*n* = 760) received a virtual examination with delivery. In the reference mode, 88% (*n* = 338) of cases received a virtual examination and an in-person examination with delivery. The proportions of cases that received a virtual examination alone (no in-person examination or delivery) because of hospital referral or workflow constraint (e.g., caller was outside delivery zone) were similar between the scalable (9%, *n* = 93) and reference (12%, *n* = 44) modes.

**Table 1:** Characteristics of participants and cases enrolled in the scalable (Improving Nighttime Access to Care and Treatment 3-H) and reference (Improving Nighttime Access to Care and Treatment 2-H) modes

Characteristic	Scalable Mode* (*n* = 1,043)^†^	Reference Mode^‡^ (*n* = 382)^§^
Participant age, median (Q1–Q3)^‖^	24 (12–48)	24 (9–48)
<2 months	15 (1%)	26 (7%)
2 months to <2 years	417 (40%)	164 (43%)
2 to <5 years	374 (36%)	118 (31%)
5–10 years	237 (23%)	74 (19%)
Participant sex, female	496 (48%)	178 (47%)
Participant chief complaint^¶^		
Fever	342 (33%)	159 (42%)
Respiratory	247 (24%)	67 (17%)
Gastrointestinal	109 (10%)	58 (15%)
Dermatologic	237 (23%)	56 (15%)
Other	108 (10%)	42 (11%)
Participant complaints general^¶^^,^^#^		
Fever	576 (55%)	212 (55%)
Respiratory	686 (66%)	247 (65%)
Gastrointestinal	337 (32%)	137 (36%)
Dermatologic	383 (37%)	100 (26%)
Case severity at call center		
Mild	788 (76%)	278 (73%)
Moderate	214 (21%)	80 (21%)
Severe	41 (4%)	24 (6%)
Type of care provided		
Virtual examination and in-person examination with delivery	190** (18%)	338 (88%)
Virtual examination with delivery	760 (73%)	0
Virtual examination alone	93 (9%)	44 (12%)

Q = quartile.

* Scalable mode was conducted in Gressier and Les Cayes.

^†^ Twenty-five repeat patients within 30 days were excluded.

^‡^ Reference mode was conducted in Gressier.

^§^ Nine repeat patients within 30 days were excluded.

^‖^ (Q1–Q3) = quartile 1 to quartile 3.

^¶^ The four most common complaints are represented.

^#^ Multiple complaints per participant were permitted.

** Nineteen mild cases and three moderate cases were triaged for delivery alone but received in-person examinations.

### Primary outcomes.

In the scalable mode, the rate of participants with a clinical status of improved/recovered was 97% (*n* = 897), and in the reference mode, the rate was 95% (*n* = 329). No mortalities occurred in the scalable mode, and one occurred in the reference mode. At the 10-day follow-up, 15% (*n* = 138) of participants in the scalable mode had sought care, and 24% (*n* = 82) of participants in the reference mode had sought care (Table 2).

**Table 2:** Comparison of clinical and operational outcomes between the scalable (Improving Nighttime Access to Care and Treatment 3-H) and reference (Improving Nighttime Access to Care and Treatment 2-H) modes

Outcomes	Scalable Mode (*n =* 930)*	Reference Mode (*n =* 348)^†^	*P*-Value
Clinical status at 10-day follow-up*			
Improved/recovered	897 (97%)	329 (95%)	0.0374^‡^
Same	26 (3%)	18 (5%)	
Worse	6 (1%)	1 (<1%)	
Care sought during 10-day follow-up period (any)	138 (15%)	82 (24%)	0.0002^§^
Type of care sought			
Hospital	75 (54%)	21 (26%)	<0.0001^‖^
Clinic	63 (46%)	55 (67%)	
Other	0	6 (7%)	
Mortalities	0	1 (<1%)	
Feedback on the TMDS service at the 10-day follow-up			
Great	859 (92%)	300 (86%)	0.0022^‡^
Good	68 (7%)	44 (13%)	
Okay	3 (<1%)	2 (1%)	
Virtual examination duration, median minutes (Q1–Q3)^¶^	16 (12–20)	20 (15–25)	<0.0001^#^
Time to arrival at household, median minutes (Q1–Q3)^¶^	78 (62–104)	73 (56–102)	0.1722^#^

Q = quartile; TMDS = telemedicine and medication delivery service.

* One hundred and thirteen participants were not reached at follow-up.

^†^ Thirty-four participants were not reached at follow-up.

^‡^ The Cochran–Armitage trend test.

^§^ The χ^2^ test.

^‖^ The Fisher exact test.

^¶^ (Q1–Q3) = quartile 1 to quartile 3.

^#^ The two-sample *t-*test.

### Secondary outcomes.

The median call duration was 16 minutes (IQR: 12–20 minutes) in the scalable mode and 20 minutes (IQR: 15–25 minutes) in the reference mode. The median time to household arrival was 73 minutes (IQR: 56–102 minutes) in the scalable mode and 78 minutes (IQR: 62–104 minutes) in the reference mode.

The aggregate rate of guideline deviations was 5% (*n* = 63) in the scalable mode and 11% (*n* = 82) in the reference mode (Table 3). Rates of missed danger signs were 1% (*n* = 11) in the scalable mode compared with 2% in the reference mode; a misinterpreted respiratory rate was the most frequent danger sign deviation. Events of medications provided (most commonly, amoxicillin) that were not indicated occurred in 1% (*n* = 18) of cases in the scalable mode and 3% (*n* = 19) of cases in the reference mode. Events of indicated medications (most commonly, zinc) that were not provided occurred in 3% (*n* = 34) of cases treated in the scalable mode and 7% (*n* = 52) of cases in the reference mode.

**Table 3:** Comparison of guideline deviations between the scalable (Improving Nighttime Access to Care and Treatment 3-H) and reference (Improving Nighttime Access to Care and Treatment 2-H) modes

Type of Guideline Deviation	Scalable Mode (*n =* 1,233)*	Reference Mode (*n =* 720)*	*P*-Value
Total deviations (all types)	63 (5%)	82 (11%)	<0.0001^†^
Danger sign present but not identified (any)	11 (1%)	11 (2%)	0.1991^†^
General^‡^	0	1 (<1%)	0.0262^§^
Fever (<2 years)	0	2 (<1%)	
Respiration rate	4 (<1%)	6 (1%)	
Heart rate	7 (1%)	1 (<1%)	
Oxygen saturation^‖^	0	1 (<1%)	
Provided medication^¶^ when not indicated (any)	18 (1%)	19 (3%)	0.0652^†^
Amoxicillin	9 (1%)	17 (2%)	0.0008^§^
Benzyl benzoate	0	0	
Cephalexin	0	0	
Doxycycline	0	0	
Erythromycin	0	2 (<1%)	
Paracetamol	7 (<1%)	0	
Zinc	2 (<1%)	0	
Did not provide medication^¶^ when indicated (any)	34 (3%)	52 (7%)	<0.0001^†^
Amoxicillin	0	2 (<1%)	0.0095^§^
Benzyl benzoate	2 (<1%)	0	
Cephalexin	0	0	
Doxycycline	0	0	
Erythromycin	3 (<1%)	0	
Paracetamol	0	0	
Zinc	29 (3%)	50 (7%)	

* Individual virtual encounters and individual in-person encounters are represented independently.

^†^ The χ^2^ test.

^‡^ General includes lethargic, convulsions, and unable to drink/breastfeed.

^§^ The Fisher exact test.

^‖^ Oxygen saturation was evaluated during in-person examinations only.

^¶^ The most frequently used medications are included.

In the scalable mode, congruence was evaluated for the subset of cases that had paired virtual and in-person examinations. In this context, the Cohen’s kappa value for cases virtually triaged as mild was minimal at 0.22 (95% CI: 0.07–0.38) (Supplemental Table 1); in the reference mode, it was 0.78 (95% CI: 0.69–0.87). The kappa value for cases virtually triaged as moderate was 0.25 (95% CI: 0.10–0.41); in the reference mode, it was 0.78 (95% CI: 0.69–0.87). A similar approach was taken to evaluate select components of the virtual examination. The assessment of fever had a kappa value of 0.24 (95% CI: 0.12–0.35); in the reference mode, the corresponding kappa value was 0.58 (95% CI: 0.49–0.66). The assessment of fast breathing was not congruent with measured rates during the in-person examination; minimal congruence was observed in the reference mode. The assessment of dehydration during the virtual examination had minimal agreement (kappa: 0.24; 95% CI: 0.01–0.47); in the reference mode, there was moderate agreement (kappa: 0.69; 95% CI: 0.41–0.98). Despite low congruence, aspects of the virtual examination had high performance metrics (sensitivity, specificity, PPV, and NPV) of over 90%. Agreement between medication recommendations based on paired virtual and in-person examinations was also assessed. In the scalable mode, paracetamol and amoxicillin exhibited minimal (kappa: 0.35; 95% CI: 0.22–0.48) and weak (kappa: 0.47; 95% CI: 0.35–0.58) agreement, respectively. Benzyl benzoate (kappa: 0.66; 95% CI: 0.49–0.83), cephalexin (kappa: 0.62; 95% CI: 0.38–0.85), and zinc (kappa: 0.62; 95% CI: 0.48–0.75) exhibited moderate congruence. These measures were consistently lower in the scalable mode than in the reference mode.

### Feedback.

Participants from the scalable mode rated the service as “great” (highest Liker-scale response) at a rate of 92% (*n* = 859) compared with 86% (*n* = 300) in the reference mode (Table 2).

### Exploratory.

Stratified analyses were performed for both the scalable and reference modes as an exploratory step to evaluate how the type of care provided (virtual examination alone; virtual examination and in-person examination with delivery; and virtual examination with delivery) impacted primary and secondary outcome measures. Within both modes, participants who received virtual examinations with delivery (with or without in-person examinations) had similar rates of improvement/recovery, care seeking, and feedback assessed as “great.” The relatively few cases in both the scalable and reference modes who received virtual examinations alone without delivery had less favorable outcomes for clinical status, care seeking, and feedback at 10 days (Table 4). Given that the scalable mode included a second delivery zone, we conducted analyses to compare outcome measures between the two sites. Clinical status, rates of care seeking, and feedback at the 10-day follow-up were similar between the delivery zones (Supplemental Table 2). However, cases in Les Cayes more commonly sought follow-up care from a hospital and experienced a longer median time to household arrival than Gressier cases.

**Table 4:** Comparison of clinical and operational outcomes stratified by type of care provided between the scalable (Improving Nighttime Access to Care and Treatment 3-H) and the reference (Improving Nighttime Access to Care and Treatment 2-H) modes

Outcomes	Scalable Mode			Reference Mode			*P*-Value
	Virtual Examination and In-Person Examination with Delivery (*n* = 167)	Virtual Examination with Delivery (*n* = 684)	Virtual Examination Alone (*n* = 79)	Virtual Examination and In-Person Examination with Delivery (*n* = 304)	Virtual Examination with Delivery (*n* = 0)	Virtual Examination Alone (*n* = 44)	
Clinical status at 10-day follow-up							
Improved/recovered	161 (96%)	666 (97%)	70 (89%)	289 (95%)	–	40 (91%)	0.2714*
Same	4 (2%)	14 (2%)	8 (10%)	14 (5%)	–	4 (9%)	
Worse	1 (1%)	4 (1%)	1 (1%)	1 (<1%)	–	0	
Care sought during 10-day follow-up period (any)	21 (13%)	76 (11%)	44 (55%)	53 (17%)	–	30 (68%)	0.471*
Type of care sought							
Hospital	12 (7%)	37 (5%)	26 (33%)	13 (4%)	–	8 (18%)	<0.0001*
Clinic	9 (5%)	36 (5%)	18 (23%)	35 (12%)	–	20 (45%)	
Other	–	–	–	4 (1%)	–	2 (5%)	
Mortalities	0	0	0	1 (<1%)	–	0	–
Feedback on TMDS service at 10-day follow-up							
Great	160 (96%)	631 (92%)	68 (86%)	273 (90%)	–	27 (63%)	0.0005*
Good	7 (4%)	51 (7%)	10 (13%)	29 (10%)	–	15 (35%)	
Okay	0	2 (<1%)	1 (1%)	1 (<1%)	–	1 (2%)	
Virtual examination duration, median minutes (Q1–Q3)^†^	18 (14–21)	15 (12–17)	17 (10–20)	20 (15–25)	–	20 (15–24)	<0.0001^‡^
Time to arrival at household, median minutes (Q1–Q3)^†^	81 (65–108)	76 (61–103)	–	73 (56–102)	–	–	0.0892^‡^

Q = quartile; TMDS = telemedicine and medication delivery service.

* The Cochran–Mantel–Haenszel test.

^†^ (Q1–Q3) = quartile 1 to quartile 3.

^‡^ Two-way analysis of variance.

## DISCUSSION

We compared a scalable mode of a TMDS that reserved in-person examinations for nonsevere cases with higher clinical uncertainty with a reference mode that required in-person examinations for all nonsevere cases; severe cases in both modes were referred to hospital-level care. We hypothesized that this maneuver would not compromise safety, with the primary outcome measures being clinical status and the rate of in-person care seeking at 10 days. To test this hypothesis, pediatric patients were prospectively enrolled in two cohort studies at different time periods to evaluate and compare the two modes. In the context of a 5-fold reduction of in-person examinations between the scalable and reference modes, rates of improvement/recovery and in-person care seeking at 10 days were similar for the scalable mode. Despite limitations, the findings support the hypothesis. Furthermore, the primary clinical findings were complemented by similar operational and feedback metrics, collectively representing a critical step toward a more scalable solution to improve early access to care without compromising safety.

The implementation of the INACT studies in Haiti required an adaptative approach to real-world events (e.g., natural disasters, political collapse, insecurity). The adaptive approach in some ways increased the generalizability and relevance of the study series to other communities, both within Haiti and globally, facing similar challenges. With this mindset, the design of the reference study required in-person examinations at households for all nonsevere cases as a clinical “guardrail.” This requirement enabled the reference study (INACT2-H) to focus on feasibility, safety, and guideline development/validation.^26^^,^^27^ In this context, we used congruence analyses to evaluate the paired virtual and in-person examinations. The analyses exposed anticipated limitations with virtual examinations that might leave providers with higher or lower clinical uncertainty depending on the clinical scenario. The clinical guidelines were developed to accommodate for this challenge. As mentioned above, our guiding hypothesis was that safety would not be compromised if the guardrail of in-person examinations was removed for cases with low clinical uncertainty and maintained for cases with higher clinical uncertainty. The results support this hypothesis, thus marking a critical step toward scalability.

Among cases with higher clinical uncertainty that required in-person examinations in the scalable mode, there was minimal/weak congruence between the virtual and in-person examinations. This finding contrasts the reference study where congruence was higher. The difference was expected as the paired cases in the scalable mode had a higher degree of clinical uncertainty than those in the reference mode by design. Taken together, the results support the need for continued in-person examinations for cases with higher clinical uncertainty. Exploratory analyses characterized the subset of participants who received a virtual examination with delivery because this group received less contact with a provider compared with those that received a virtual examination and an in-person examination with delivery. Similar clinical outcomes and feedback scores were observed between these two groups. These data suggest a noninferior patient experience for those cases that received virtual examinations with delivery. The small subset of participants who received a virtual examination alone (no in-person encounter by a provider or delivery driver) may have had inferior outcomes in terms of clinical status and feedback at 10 days. This finding is not unexpected considering that these participants received advice only without provided medication.

Throughout the INACT studies, we have pushed back against a hesitancy captured in a prior survey that found that less than half of Haitian health care providers believed that telemedicine could serve as an alternative to in-person care in mitigating the impacts of gang violence on health care provision in Haiti.^38^ Rather, we advocate that an innovative design built iteratively over time with broad stakeholder input can yield a resilient and hopefully effective solution. One “real-world” example from this study was an earthquake that occurred southwest of the Gressier site soon after initiating the scalable mode.^39^ For humanitarian reasons, a delivery zone in Les Cayes was added, which required an additional step of relaying virtual treatment plans from the Gressier call center to on-call providers in Les Cayes. This adaptation could represent a potential study limitation; however, stratification by study site yielded similar outcomes between the two delivery zones. The rate of care seeking at the hospital level was higher in Les Cayes, likely because of increased hospital access within the delivery zone. Les Cayes participants also experienced a longer median time to household arrival that was likely caused by the need to mobilize the on-call provider; the road network in Les Cayes is better and may have mitigated the extent of the longer arrival time.

The results from this study should be viewed within the context of the following additional limitations. 1) Virtual treatment plans only addressed complaints that parents chose to report. In contrast, in-person treatment plans addressed all problems identified by the provider, allowing virtual and in-person plans to be based on different problems. This factor limited the treatment plan congruence analyses. 2) The clinical resources have undergone minor iterations throughout both study periods. Therefore, the treatment plans were not absolutely equivalent at all points. In addition, we did not control for increased provider expertise over the study period. 3) The environment in which both studies were conducted was volatile, which may have confounded enrollment, operational outcomes, and disease severity over time. 4) The primary outcome measures were based on parent reports from the 10-day follow-up call, and therefore, they are at risk of response and recall bias. 5) The sample size was low, and stratified analyses to assess important variables such as age and sex, even on an exploratory basis, were not possible. Despite these limitations, the study provides evidence to justify a transition to the scalable mode.

## CONCLUSION

Early access to health care is essential to reduce morbidity and mortality. However, innovative strategies are needed to assure early access to care, especially when patients are isolated by geography, poverty, or illness that presents “off-hours.” A TMDS represents an innovative solution to extend care to these vulnerable populations. We evaluated a scalable design for a pediatric TMDS for low-resource settings that triaged severe cases to hospital-level care, nonsevere cases with higher clinical uncertainty to in-person examinations at households with medication delivery, and nonsevere cases with low clinical uncertainty to medication delivery alone. We found that within the context of a 5-fold reduction of in-person examinations, participants in the scalable mode had noninferior rates of improvement/recovery and care seeking. Future steps toward scaling will be to digitize the TMDS clinical resources, implement this digital tool within the scalable TMDS workflow, and evaluate outcomes.

## Supplemental Materials

10.4269/ajtmh.24-0846 Supplemental Materials
