# Supplementary material for: Evaluation of a Scalable Design for a Pediatric Telemedicine and Medication Delivery Service: A Prospective Cohort Study in Haiti
Source: Am J Trop Med Hyg. 2025 Jun 24;113(3):704–13. doi: 10.4269/ajtmh.24-0846 (PMC12410253; doi:10.4269/ajtmh.24-0846)
Supplement: Supplemental Materials [file tpmd240846.SD1.pdf]

Supplementary Materials

Evaluation of a scalable design for a pediatric telemedicine and medication delivery service: A prospective cohort study in Haiti

S1 Table. Congruence between select components of virtual and in-person exams from the scalable (INACT3-H) mode. 2

S2 Table. Comparison of clinical and operational outcomes of the scalable mode (INACT3-H) stratified by delivery zone 3

S1 Table. Congruence between select components of virtual and in-person exams from the scalable (INACT3-H) mode.

		Scalable mode									
Component		Total	CC+ HH+ (%)	CC+ HH- (%)	CC- HH+ (%)	CC- HH- (%)	Sens. (95% CI)	Spec. (95% CI)	PPV (95% CI)	NPV (95% CI)	Kappa (95% CI)
Severity	Mild	173	96 (55)	23 (13)	32(19)	22(13)	75(68-83)	49(34-63)	81(74-88)	41(28-54)	0.22(0.07-0.38)
	Moderate	173	22 (13)	32 (19)	20(16)	99(57)	52(37-67)	76(68-83)	41(28-54)	83(76-90)	0.25(0.10-0.41)
Vital signs	Fever	189	91 (48)	62 (33)	8(4)	28(15)	92(87-97)	31(22-41)	59(52-67)	78(64-91)	0.24(0.12-0.35)
	Fast breathing	58	0 (0)	4 (7)	3 (6)	51(88)	0(0-0)	93(86-100)	0(0-0)	94(88-100)	-0.06(-0.11- -0.01)
Dehydration assessment	None	65	40 (62)	3 (5)	16(25)	6(9)	71(60-83)	67(36-97)	93(85-100)	27(9-46)	0.24(0.01 -0.47)
	Moderate	65	6 (9)	16 (25)	3(5)	40(62)	67(34-97)	71(60-83)	27(9-46)	93(85-100)	0.24(0.01 -0.47)
Medications in treatment plan	Amoxicillin	192	72 (38)	45 (23)	8(4)	67(35)	90(83-97)	60(51-69)	62(53-70)	89(82-96)	0.47(0.35-0.58)
	Benzyl benzoate	192	15 (8)	2 (1)	11(6)	164(85)	58(39-77)	99(97-100)	88(73-100)	94(90-97)	0.66(0.49-0.83)
	Cephalexin	192	8 (4)	5 (3)	4(2)	175(91)	67(40-93)	97(95-100)	62(35-88)	98(96-100)	0.62(0.38-0.85)
	Paracetamol	192	115 (60)	44 (23)	7(4)	26(14)	94(90-98)	37(26-48)	72(65-79)	79(65-93)	0.35(0.22-0.48)
	Zinc	192	29 (15)	6 (3)	19(10)	138(72)	60(47-74)	96(93-99)	83(70-95)	88(83-93)	0.62(0.48-0.75)

CC= call center, HH= household, '+' = present, '-' = absent, PPV = positive predictive value, NPV= negative predictive value, Kappa = Cohen’s kappa

S2 Table. Comparison of clinical and operational outcomes of the scalable mode (INACT3-H) stratified by delivery zone.

	All (N=855[a])	Gressier (n= 314[a])	Les Cayes (n=541[a])	*P*-value
Clinical status at 10-day follow-up				.3687[b]
Improved/Recovered	830 (97%)	304 (97%)	526 (97%)	
Same	19 (2%)	8 (3%)	11 (2%)	
Worse	5 (<1%)	1 (<1%)	0	
Care sought by 10-day follow-up (any)	94 (11%)	36 (11%)	58 (11%)	.7275[c]
Type of care sought at 10 days				
Hospital	48 (51%)	9 (25%)	39 (67%)	.0003[c]
Clinic	46 (49%)	27 (75%)	19 (33%)	
Mortalities	0	0	0	
Feedback on TMDS service at 10-day follow-up				.1270[b]
Great	795 (93%)	297 (95%)	498 (92%)	
Good	58 (7%)	17 (5%)	41 (8%)	
Okay	2 (<1%)	0	2 (<1%)	
Virtual exam duration (median minutes, Q1-Q3)[e]	17 (12-21)	18 (14-23)	16 (12-20)	<.000[d]
Time to arrival at household (median minutes, Q1-Q3)[e]	78 (62-104)	72 (59-99)	81 (64-110)	<.000[d]

[a] Includes participants who received a delivery with or without in-person exam and who were reached at 10-day follow-up.
[b] Cochran-Armitage Trend Test.
[c] Chi- Square Test.
[d] Two-Sample T Test.
[e] (Q1-Q3) = quartile 1 to quartile 3.
